# The impact of socioeconomic status on changes in the general and mental health of women over time: evidence from a longitudinal study of Australian women

**DOI:** 10.1186/1475-9276-12-25

**Published:** 2013-04-09

**Authors:** Jennifer Stewart Williams, Michelle Cunich, Julie Byles

**Affiliations:** 1 Research Centre for Gender Health & Ageing, Faculty of Health, University of Newcastle, HMRI Building, Callaghan, NSW, 2308, Australia; 2 Sydney School of Public Health Room 314, A-27 Edward Ford Building University of Sydney, Sydney, NSW, 2006, Australia

**Keywords:** Age, Aging, Women, Health inequalities, Socioeconomic, Health change, Mental health

## Abstract

**Introduction:**

Generally, men and women of higher socioeconomic status (SES) have better health. Little is known about how socioeconomic factors are associated with changes in health as women progress through mid-life. This study uses data from six survey waves (1996 to 2010) of the Australian Longitudinal Study on Women’s Health (ALSWH) to examine associations between SES and changes in the general health and mental health of a cohort of women progressing in years from 45–50 to 59–64.

**Methods:**

Participants were 12,709 women (born 1946–51) in the ALSWH. Outcome measures were the general health and mental health subscales of the Medical Outcomes Study Short Form 36 Questionnaire (SF-36). The measure of SES was derived from factor analysis of responses to questions in the ALSWH baseline survey (1996) on school leaving age, highest qualifications, and current or last occupation.

Multi-level random coefficient models, adjusted for socio-demographic factors and health behaviors, were used to analyze repeated measures of general health and mental health. Survey year accounted for changes in factors across time. In the first set of analyses we investigated associations between the SES index, used as a “continuous” variable, and general health and mental health changes over time. To illuminate the impact of different levels of SES on health, a second analysis was conducted in which SES scores were grouped into three approximately equal sized categories or “tertiles” as reported in an earlier ALSWH study. The least square means of general and mental health scores from the longitudinal models were plotted for the three SES tertiles.

**Results:**

The longitudinal analysis showed that, after adjusting for the effects of time and possible confounders, the general (mental) health of this cohort of mid-aged women declined (increased) over time. Higher SES women reported better health than lower SES women, and SES significantly modified the effects of time on both general and mental health in favor of higher SES women.

**Conclusions:**

This study contributes to our current understanding of how socioeconomic and demographic factors, health behaviors and time impact on changes in the general and mental health of women progressing in years from 45–50 to 59–64.

## Introduction

Generally, people of higher socioeconomic status (SES) have better health indicators [1]. Poor education, low income, adverse living and working conditions, and stress are among the social and economic factors or “social determinants” [2] normally associated with poor health. Many areas of health policy focus on either reducing an unfair distribution of healthcare, or assisting individuals in overcoming avoidable health inequalities [3].

Changes in health occur with advancing age. This is attributable not only to biology but also to social and economic factors. Globally, both in absolute and relative terms, people are living longer but they are also spending more years in declining health [4]. The ageing of our populations and socioeconomic inequalities in health present major challenges for many areas of policy [5,6].

Understanding associations between SES and health changes with ageing is important for the development of polices aimed at reducing health inequalities. For example, if there are phases in the life course when social and economic factors have a major impact on health decline, then policies may be more effective if targeted at these specific ages, as well as socioeconomic groups, where there are identifiable health gains to be made. Cross-sectional studies cannot assess the impact of SES on health changes over time. Longitudinal cohort studies, on the other hand, can disentangle the associations between age, cohort, sex and socioeconomic inequalities in health over time with advancing age. Evidence from these studies can assist policy-makers in the development, targeting and timing of interventions to reduce health inequalities that accumulate across the life-span [7-13]. However the success of such policies will be influenced by knowledge of the specific times of life at which people are more vulnerable to the negative impacts of social, economic and other factors on their health [14].

There is epidemiological evidence that socioeconomic inequalities in health are age-dependent. Several life course models that incorporate health as a time-varying or age-related variable show that inequalities in health widen in mid-age as a consequence of accumulated social and economic disadvantage [8,15-17]. But studies have also demonstrated that there is convergence of health inequalities in older age [18,19] because morbidity is compressed among more affluent groups until late-life [20]. One explanation for this is that disadvantaged people die younger, resulting in a smaller gap in health inequalities among people who survive to older age [18]. Yet other research has shown that “selective” survival on its own does not fully explain the compactness of health inequalities in later life [21,22]. A further reason for a narrowing of health inequalities in older age may be found in the welfare systems of countries. In Australia, Canada and the United Kingdom, for example, health service use is more heavily subsidized for older people, thereby reducing socioeconomic differentials in access to health and medical services and medications in older age groups [23,24].

Numerous studies have demonstrated that health differences are mediated by gender and age. Socioeconomic factors (e.g. income and financial independence) have repeatedly been shown to have an impact on the health of women in mid-age [25-28]. In addition, studies have demonstrated that, compared to men, women experience greater health disadvantage which is amplified by SES [29]. However, little is known about the impact of socioeconomic factors on health change as women progress through mid-life.

### Aims

This study uses self-reported health survey data from a cohort of community-dwelling women (born 1946–1951) in the Australian Longitudinal Study on Women’s Health (ALSWH). A previous ALSWH study, using two survey waves, showed a widening of SES differentials in physical health for mid-aged women (born 1946–1951) between 1996 and 1998, and a narrowing for older-aged women (born 1921–1926) between 1996 and 1999 [23]. This study uses six survey waves of ALSWH data (1996–2010). The main aim is to examine associations between SES and changes in the general and mental health of a cohort of women progressing in years from 45–50 to 59–64.

## Methods

### Study population

The ALSWH is a national twenty-year prospective cohort study of changes in the health and well-being of Australian women born 1973–1978, 1946–1951 and 1921–1926 [30]. Samples from each age cohort were randomly selected from the Medicare Australia national insurance database [31]. In this paper, participants were from the cohort of women in ALSWH who were born 1946–51, which we refer to as the “mid-aged cohort”. These women were surveyed (using mailed questionnaires) at baseline in 1996 when they were aged 45–50 years, and subsequently in 1998, 2001, 2004, 2007 and 2010 [32,33]. Data collected at each of these six survey time-points are analyzed in this paper.

Information on study methods, representativeness, women’s characteristics and response rates is given at http://www.alswh.org.au/. The University of Newcastle Human Research Ethics Committee approved all aspects of the study (H-076-0795).

### Measures of health

Health-related quality of life (HRQL) was measured at each survey time-point using the Australian version 1 of the Medical Outcomes Study Short Form 36 (SF-36) [34]. The SF-36 is a validated self-report measure of HRQL. It consists of 36 items relating to eight different health domains. At each survey, and for each health domain (or subscale), a weighted sum of responses to items is calculated to derive a score between 0 (lowest well-being) to 100 (highest well-being) [35]. Both the general and mental health subscales of the SF-36 were used for the analyses of in this paper to compare/contrast two different domains of HRQL.

### Measures of SES

The key independent variable in the longitudinal data models was SES. The measure of SES used here was developed specifically for women in the ALSWH mid-aged cohort. An index was derived from factor analysis of ALSWH data using the method of principal components with varimax rotation [36,37]. Responses to questions on school leaving age, highest qualifications and current or last occupation asked in Survey 1 were included because these items showed strong associations with the health of women in the ALSWH mid-aged cohort [37]. The factor weights were as follows: (i) *age first left school*: 16 years or younger (1); 17 years or older (2); (ii) *highest qualification attained*: no formal qualification (1); school certificate (2); higher school certificate (3); trade, apprenticeship, certificate or diploma (4); higher degree or bachelor degree (5), (iii) *occupation*: never had a paid job (1); machine operator, cleaner or similar (2); advanced/intermediate sales, clerk or personal service worker (3); associate professional or trades-person (4); manager or administrator, professional (5). Responses to these questions were weighted and summed to derive SES scores (integers), with higher values indicating greater socioeconomic advantage. The scores ranged from 3 to 12 with a mean of 7.6 and a median of 7.0.

In the first set of analyses we investigated associations between changes over time in general health and mental health by SES, using the SES index as a “continuous” variable. In these analyses associations were averaged across the range of SES values (i.e. 3 to 12). In the second set of analyses, women were ranked by their SES scores to create three categories, or “tertiles” as reported in the earlier ALSWH study [23]. The lowest tertile (least advantaged) comprised scores 3 to 6, the middle tertile comprised scores 7 to 9, and the highest (most advantaged) tertile comprised scores 10 to 12. We investigated differential changes over time in the general and mental health of women across these SES tertiles.

### Covariates

The time covariate was represented by a dummy variable that identified survey year. All models were adjusted for potential socio-demographic and health-related confounders measured at Survey 1. These were: age in years (45, 46, 47, 48, 49 and 50); area of residence; marital status; body mass index (BMI); smoking; ability to manage on income, and stress. Area of residence was a binary classification: urban versus rural or remote. Marital status was classified as married or de-facto versus separated, divorced, widowed, or single. Body mass index was categorized as: underweight (BMI<18.5); healthy weight (18.5<=BMI<25); overweight (25<=BMI<30) and obese (BMI>=30). Smoking status was categorized as: non-smoker; ex-smoker and smoker. Ability to manage on income was categorized as: impossible or difficult always; difficult sometimes; not too bad and easy. Stress was represented as a continuous variable with higher scores indicating greater personal stress. For a detailed description and definition of these survey variables see http://www.alswh.org.au/.

### Statistical analysis

Multi-level random coefficient models were used to analyze repeated measures of general health and mental health for women at the six survey time points. Survey year (time) was included in all models to account for changes in general and mental health over time. The models were also adjusted for the effects of SES, age (measured at baseline), area of residence, marital status, BMI, smoking status, ability to manage on income and stress.

Four longitudinal models were constructed. In models 1 and 2, the ungrouped SES scores were used to investigate averaged associations between SES and general and mental health. In models 3 and 4, the SES tertiles were used to investigate differential changes in health.

Longitudinal analyses were performed using the mixed procedure for multi-level modeling in SAS version 9.1. After checking appropriateness, an unstructured covariance structure was used. The Maximum Likelihood Method compared goodness of fit [38].

## Results

### Population characteristics

Table 1 shows socio-demographic and health-related characteristics of ALSWH women in the 1946–51 cohort at survey 1 (1996) by low, middle (mid) and high SES categories (n=12,709). Of these women, 38.9% were in the low SES tertile, 35.6% were in the mid SES tertile and 25.5% were in the high SES tertile. Compared with low and mid SES groups, a greater proportion of high SES women were living in urban areas (78.0% vs. 69.1% vs. 71.8%); separated, divorced, widowed or single (21.8% vs. 16.6% vs. 18.9%); non-smokers (58.5% vs. 51.4% vs. 51.3%); had normal body weight (59.7% vs. 49.3% vs. 52.9%), and found it easy to manage on their incomes (22.8% vs. 12.6% vs. 14.6%).

**Table 1:** **Socio**-**demographic and health**-**related characteristics of women in the 1946**–**51 ALSWH**^**1 **^**cohort at survey 1 in 1996** (**N**=**12**,**709**)

	**Low SES (n=4,943) %**	**Mid SES (n=4,531) %**	**High SES (n=3,235) %**	**Total (n=12,709) %**
**Area of residence**				
Capital city/Other metropolitan centers	69.1	71.8	78.0	72.6
Large/Small rural centers or remote	30.9	28.2	22.0	27.4
**Marital status**				
Married/De-facto	83.4	81.2	78.2	81.1
Separated/Divorced/Widowed/Single	16.6	18.9	21.8	18.9
**Smoker**				
Non-smoker	51.4	51.3	58.5	53.4
Ex-smoker	26.9	30.8	29.6	29.0
Smoker	21.7	17.8	11.9	17.6
**Body Mass Index** (**BMI**)				
Normal weight (18.5-24.9 kg/m^2^)	49.3	52.9	59.7	53.6
Underweight (< 18.5 kg/m^2^)	1.5	1.8	2.6	1.9
Overweight (25.0-30.0 kg/m^2^)	28.8	28.0	25.8	27.6
Obese (Obese (>30.0 kg/m^2^)	20.5	17.3	11.9	16.9
**Ability to manage on income**				
Easy	12.6	14.6	22.8	16.2
Not too bad	40.6	43.6	43.3	42.4
Difficult sometimes	30.2	27.5	23.6	27.4
Impossible or difficult always	16.7	14.3	10.4	14.1
**Age first left school**				
16 years or younger	56.7	36.0	7.3	67.1
17 years or older	2.6	34.9	62.7	32.9
**Highest qualification attained**				
No formal qualification	88.1	11.9	0.0	17.2
School certificate	70.3	29.7	0.0	31.6
Higher school certificate	8.8	79.0	12.1	16.9
Trade, apprenticeship, certificate or diploma	0.0	52.0	48.0	19.8
Higher degree or bachelor degree	0.0	4.0	96.0	14.5
**Main occupation** (**current or last**)				
Never had a paid job	92.7	7.3	0.0	1.6
Machine operator, cleaner	87.0	13.1	0.0	15.3
Advanced/Intermediate sales, clerk, personal service worker	57.0	41.5	1.5	40.4
Associate professional or trades-person	8.0	57.2	34.9	13.1
Manager/Administrator/Professional	0.0	31.5	68.6	29.6
**Total**	38.9	35.6	25.5	100.00

^1^ Australian Longitudinal Study on Women’s Health.

Data are weighted to adjust for oversampling in rural and remote areas.

Percentages vary due to rounding.

Table 2 presents crude mean (standard deviation) SF-36 general health and mental health subscale scores at each of the six survey ALSWH time-points for women in the 1946–51 ALSWH cohort who responded to any of these surveys. The estimates show that general health decreased and mental health improved for these women between 1996 and 2010.

**Table 2:** **General health and mental health scores by ALSWH**^**1 **^**survey 1 to survey 6 for 1946**–**51 ALSWH cohort**

**Survey number and year**	**General health SF-36 sub-scale mean (SD)**^**2**^	**Number of women with general health score on SF**-**36 sub**-**scale**	**Mental health SF-36 sub-scale mean (SD)**^**2**^	**Number of women with mental health score on SF**-**36 sub**-**scale**
Survey 1: 1996	72.15 (20.47)	13,209	72.85 (17.89)	13,592
Survey 2: 1998	72.90 (20.39)	12,242	73.69 (18.47)	12,299
Survey 3: 2001	71.40 (20.66)	10,883	73.85 (18.04)	11,142
Survey 4: 2004	70.87 (20.85)	10,633	74.66 (17.96)	10,849
Survey 5: 2007	71.29 (20.67)	10,386	75.77 (17.59)	10,592
Survey 6: 2010	70.39 (20.55)	9,988	76.49 (17.47)	9,988

^1^ Australian Longitudinal Study on Women’s Health.

^2^ SD standard deviation.

### Changes in general and mental health over time

Parameter estimates from Models 1 and 2, for women who responded to any of the six ALSWH surveys, are presented in Table 3. In these models, SES is a “continuous” variable with lower scores indicating lower SES.

**Table 3:** **Longitudinal estimates** (**S1**-**S6**)^**1 **^**of general health and mental health by SES**, **1946**–**51 ALSWH**^**2 **^**cohort**

	**General health**^**3 **^**estimate model 1**	**P**-**value**	**Mental health**^**4 **^**estimate model 2**	**P**-**value**
**Intercept**	**80**.**9924**	<.0001	**76**.**2838**	<.0001
**SES**	**0**.**3408**	<.0001	**0**.**3972**	<.0001
**Time** (years since Survey 1)	−**0**.**5543**	<.0001	−0.0342	0.3871
**Interaction**: SES*Time	**0**.**0371**	<.0001	**0**.**0275**	<.0001
**Age**	−0.1639	0.1127	**0**.**3801**	<.0001
**Body mass index**				
Normal	**Reference**		**Reference**	
Underweight	−**3**.**4426**	0.0040	−1.2232	0.2088
Overweight	−**3**.**2655**	<.0001	−0.2578	0.3763
Obese	−**9**.**5538**	<.0001	−**1**.**4620**	<.0001
**Marital status**				
Married/De-facto	**Reference**		**Reference**	
Divorced/Separated	−**0**.**9217**	0.0319	−**0**.**6888**	0.0487
**Smoking status**				
Non-smoker	**Reference**		**Reference**	
Ex-smoker	0.3112	0.3790	−**0**.**5994**	0.0369
Smoker	−**4**.**9801**	<.0001	−**3**.**3996**	<.0001
**Area of residence**				
Urban	**Reference**		**Reference**	
Rural	**1**.**2768**	<.0001	**2**.**0328**	<.0001
**Able to manage on income**				
Easy	**Reference**		**Reference**	
Difficult sometimes	−**2**.**8944**	<.0001	−**1**.**9830**	<.0001
Not too bad	−**6**.**0031**	<.0001	−**4**.**7913**	<.0001
Impossible/Difficult always	−**11**.**2947**	<.0001	−**10**.**1975**	<.0001
**Stress**				
Major life experiences	−**23**.**9689**	<.0001	−**29**.**9691**	<.0001

^1^ Survey 1 to Survey 6.

^2^ Australian Longitudinal Study on Women’s Health.

^3^ General health SF-36 subscale.

^4^ Mental health SF-36 subscale.

Estimates in bold type significant p< 0.05.

Model 1 shows a statistically significant (p<0.0001) decline in the general health of women over time (−0.5543). The interaction between SES and time was positive (0.0371) and statistically significant (p<0.0001). Model 1 also shows a statistically significant (p<0.0001) positive association (0.3408) between SES and general health, after adjusting for the interaction between SES and time, survey year (or time since completing survey 1), age (measured at baseline) and the women’s socio-demographic (area of residence, marital status, ability to manage on income) and health-related characteristics (smoking status, BMI, stress) which are seen as possible confounders. Age was not statistically significant. The results of Model 1 show that, on average, the general health of women declined over time. Moreover, the effect of time on general health was modified by SES, with higher SES women having better general health.

In Model 2 the association between time and mental health (−0.0342) was not statistically significant but the interaction between SES and time (0.0275) was statistically significant and positive (p<0.0001). There was a statistically significant positive association (p<0.0001) between SES and mental health (0.3972) after adjusting for the interaction between SES and time, survey year, age (measured at baseline) and the set of possible confounders noted above. The association between age (measured at baseline) and mental health was statistically significant (p<0.0001) and positive (0.3801). The results of Model 2 suggest that, on average, the women experienced improvements in mental health as they advanced in age. The effect of time on mental health was modified by SES, favouring higher SES women.

**Figure 1:**
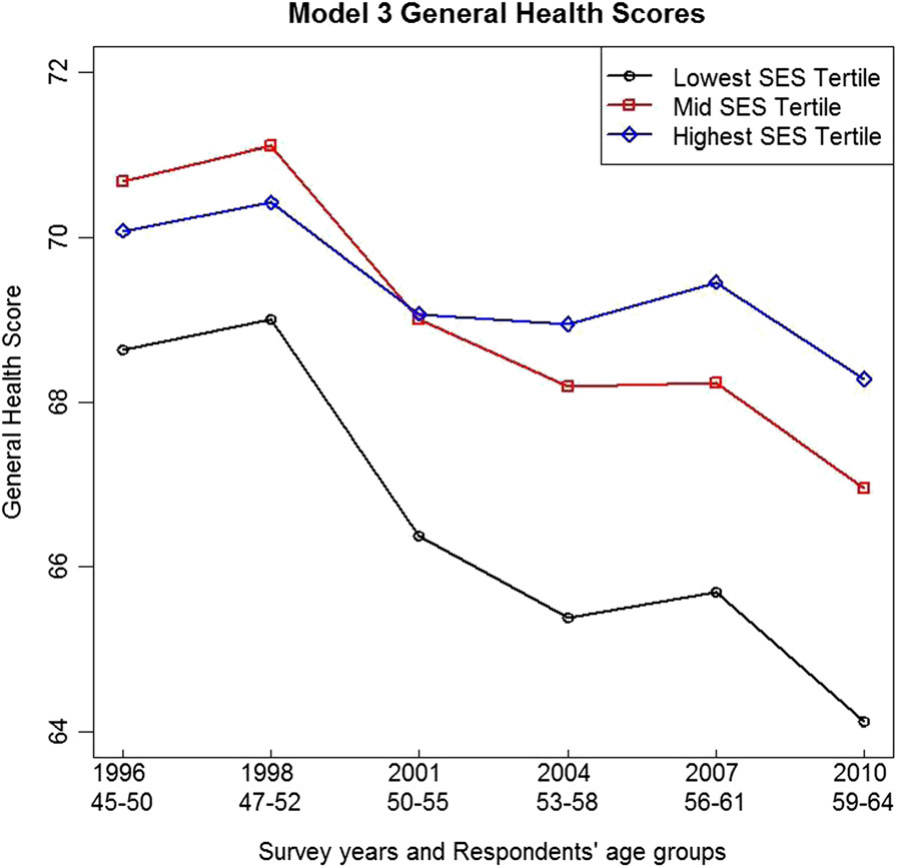
**Model 3 general health adjusted least square means across ALSWH survey years 1 to 6**, **by SES tertiles.***Note*: *Two years between ALSWH baseline Survey 1 and Survey 2 and three years between subsequent survey*s.

**Figure 2:**
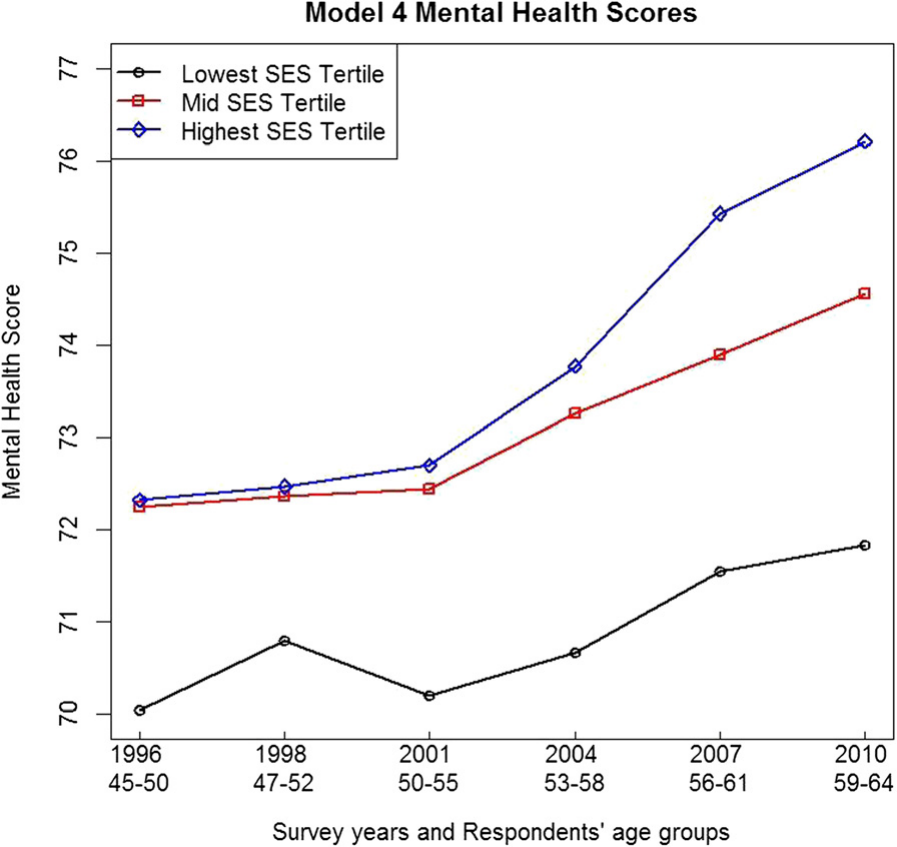
**Model 4 mental health adjusted least square means across ALSWH survey years 1 to 6, by SES tertiles.***Note*: *Two years between ALSWH baseline Survey 1 and Survey 2 and three years between subsequent survey*s.

Parameter estimates from Models 3 and 4 for women who responded to any of the six ALSWH surveys are given in Table 4. The health scores from these models are shown in Figures 1 and 2. In Models 3 and 4 SES is measured categorically in tertiles of low (reference), middle and high SES.

**Table 4:** **Longitudinal estimates** (**S1**-**S6**)^**1 **^**of general health and mental health by SES, high**/**mid**/**low categories**, **1946**–**51 ALSWH**^**2 **^**cohort**

	**General health**^**3 **^**estimate model 3**	**P**-**value**	**Mental health**^**4 **^**estimate model 4**	**P**-**value**
**Intercept**	**82**.**7605**	<.0001	**78**.**4172**	<.0001
**SES Groups**				
Low	**Reference**		**Reference**	
Mid	**2**.**1192**	<.0001	**1**.**9351**	<.0001
High	**1**.**3834**	0.0013	**1**.**7907**	<.0001
**Time** (years since Survey 1)	−**0**.**3427**	<.0001	**0**.**1128**	<.0001
**Age**	−0.1718	0.0963	**0**.**3719**	<.0001
**Interaction**: SES*Time				
Low SES*Time	**Reference**		**Reference**	
Mid SES*Time	0.0553	0.0635	0.0542	0.0468
High SES*Time	**0**.**2185**	<.0001	**0**.**1805**	<.0001
**Body mass index**				
Normal	**Reference**		**Reference**	
Underweight	−**3**.**4047**	0.0044	−1.1942	0.2200
Overweight	−**3**.**3426**	<.0001	−0.3256	0.2637
Obese	−**9**.**6763**	<.0001	−**1**.**5703**	<.0001
**Marital status**				
Married/De-facto	**Reference**		**Reference**	
Divorced/Separated	−**0**.**8448**	0.0492	−0.6304	0.0713
**Smoking status**				
Non-smoker	**Reference**		**Reference**	
Ex-smoker	0.2893	0.4135	−0.6156	.0322
Smoker	−**5**.**1002**	<.0001	−**2**.**0835**	<.0001
**Area of residence**				
Urban	**Reference**		**Reference**	
Rural	**1**.**2038**	0.0002	**1**.**9726**	<.0001
**Able to manage on income**				
Easy	**Reference**		**Reference**	
Difficult sometimes	−**3**.**0150**	<.0001	−**2**.**0835**	<.0001
Not too bad	−**6**.**1416**	<.0001	−**4**.**9092**	<.0001
Impossible/Difficult always	−**11**.**4870**	<.0001	−**10**.**3650**	<.0001
**Stress**				
Major life experiences	−**24**.**0024**	<.0001	−**30**.**0025**	<.0001

^1^ Survey 1 to Survey 6.

^2^ Australian Longitudinal Study on Women’s Health.

^3^ General health SF-36 subscale.

^4^ Mental health SF-36 subscale.

Estimates in bold type significant p< 0.05.

Model 3 shows a statistically significant decline in general health over time (−0.3427). The interaction between the high SES tertile and time (0.2185) was statistically significant and positive (p<0.0001) while the interaction between the mid SES tertile and time was not significant. This means that the decline in general health was less for women in the high SES tertile. In Model 3, note that the coefficient for the association between the high SES tertile and general health was smaller than the coefficient for the mid SES tertile and general health. This result is apparent in Figure 1 from the lower starting point for general health for women in the high compared with the mid SES tertile. 

Figure 1 shows the general health adjusted least squares means from Model 3 at each of the ALSWH survey years, by SES tertiles.

In Figure 1 the mid and low SES tertiles show falling general health over time. In the high SES tertile, general health declined more slowly, and apart from the first two surveys, was better for women in the high SES tertile than the mid and low SES tertiles. Surprisingly, Figure 1 shows a small improvement in the general health for women for all tertiles between the first two surveys (in 1996 and 1998), with women in the mid SES tertile doing better than those in the high SES tertile. Some possible reasons are suggested in the Discussion section.

Model 4 shows a statistically significant (p<0.0001) improvement in the mental health of women (0.1128). As in Model 3, the interaction between the high SES tertile and time (0.1805) was statistically significant and positive (p<0.0001). The interaction between the mid SES tertile and time (0.0542) was not significant. Improvements in mental health were greater for women in the high SES tertile.

Figure 2 shows the general health adjusted least squares means from Model 4 at each of the ALSWH survey years, by SES tertiles.

In Figure 2 the mid and low SES tertiles show improvement in mental health over time. The mental health of women in the high SES tertile improved at a faster rate compared to those women in the mid and low SES tertiles. Figures 1 and 2 show that for both general and mental health, women in the lowest SES tertile had poorer health than women in the mid and high tertiles.

All models showed that general and mental health was poorer for women who were overweight or obese, smokers, found it impossible to manage on their household income, and experienced high levels of stress. Women living in rural areas reported better general health and mental health than those women in urban areas. Stress was highly significant and negatively associated with both health measures.

## Discussion

This study reports on a longitudinal analysis of the effects of SES on the general health and mental health of women from a cohort of community-dwelling mid-aged Australian women in the ALSWH. The survey data cover the years from 1996 to 2010 during which time the women advanced in years from 45-50, to 59-64. Our analysis shows that, after adjusting for the effects of SES, time and possible confounders, the (self-reported) general (mental) health of this cohort of mid-aged women declined (increased) over time.

Socioeconomic status was measured using a validated index based on the women’s responses to items on education and current or last employment in the ALSWH baseline survey (1996). The index comprised a set of integers (3 to 12) with higher values indicating greater socioeconomic advantage. Models 1 and 2 showed averaged associations between the SES index and the changes in general health and mental health over time (1996 to 2010). However averages mask variations. In order to investigate the effects of variation in SES, and for consistency with an earlier ALSWH study [23], we also used SES tertiles to analyze these data. Models 3 and 4 showed associations between SES tertiles and changes in general health and mental health over time (1996 to 2010). Figures 1 and 2 show that women in the lowest SES tertile had poorer general and mental health compared with higher SES women.

Overall, SES significantly modified the effects of time on both the general and mental health of women, in favor of women with higher SES. The SES index was based on education and occupation. We suggest that highly educated professional women in their early and mid-fifties may have reported better health in the ALSWH surveys as a result of benefiting from programs and services such as cancer screening, counseling, physiotherapy, remedial massage, and occupational health initiatives. Suitable policy responses may include primary care, community and workplace interventions and strategies to ensure that low and mid SES women in mid-age have the same opportunities to realize health benefits as high SES women.

There was a positive and significant association between age and mental health suggesting that mental health was better for older women in this cohort. These findings are consistent with other analyses of mental health changes over time in the ALSWH 1946–51 cohort [39,40] and in other samples and/or studies [41-43]. It has been suggested that improvements in self-reported mental health reflect women’s changing expectations of their health as they age [40] and also positive psychological adjustments that follow from a reorientation of values in mid-life [39]. Another explanation is that while social and economic circumstances (e.g. job strain, sole parenthood) and biology (e.g. menopause) can have profound effects on women’s mental health in early mid-age [30,44-48], these pressures may attenuate in older aged women. Other studies have linked improved mental health in late mid-age with retirement from the workforce [26,49]. Policies aimed at improving women’s mental health must also take into account the need to target specific age as well as SES groups.

There is a need to investigate the pathways through which social, economic, and demographic factors impact positively on general and mental health changes in women as they advance through mid-age. It is important to consider potential barriers (e.g. cost, transport, health information dissemination) that may be preventing lower SES women from better managing and improving their health to the same extent as women in high SES.

Figure 1 shows that the general health of women in all three SES tertiles improved in the two years between 1996 and 1998. While this was an unexpected result, it is possible that this was due to an artifact in self-reported health at the ALSWH baseline survey. For example, women may have unassumingly under-reported their general health at baseline due to being unsure about what was expected of them in this first major national longitudinal survey of Australian women. There are a number of methodological challenges associated with follow up responses in longitudinal surveys [50] and this may be an area for further investigation.

Figure 1 also shows that women in the high SES tertile reported poorer general health compared with women in the mid SES tertile between 1996 and 2001, when they were in their late forties and early fifties. A possible explanation for this is that, compared to women in the low SES tertile, women in the high SES tertile experienced greater career and family pressures due to social expectations and high achievement aspirations that impacted negatively on their reporting of general health in the ALSWH surveys. However the findings show distinctly that there were major differences between health and health change between the mid and high SES tertiles versus the low SES tertile. Higher SES women (i.e. from the mid and high tertiles) reported better general and mental health. (See Figures 1 and 2).

The results of this study are consistent with previous studies that have demonstrated: (i) higher SES is positively associated with better self-reported health [51]; (ii) physical health declines in mid-age [52], and (iii) SES mediates health change over time [53,54]. Mid-aged women are an important sub-population with specific physical and mental health needs associated with biological, social and economic circumstances. These findings may be used to inform policy-makers about possible strategies to address undesirable socioeconomic patterning (inequalities) of general health and mental health changes in women in mid-life and beyond.

Reductions in the general health of mid-aged women are mainly attributable to biology and genetics. However this study shows that socioeconomic and behavioral factors, such as SES, BMI, marital status, smoking status, ability to manage on income and stress, significantly intensify physiological changes in general health that occur in women as they move through mid-age. In Australia almost one third of ill health associated with disability and premature death is attributed to lifestyle behaviors (e.g. tobacco use, physical inactivity), physiological states, (e.g. high body mass index, high blood pressure, high cholesterol), and social and environmental factors, such as urban air pollution and occupational exposures [55]. There is growing evidence that many of these factors can be modified by targeted policy actions, e.g. through the promotion of healthy body weight through exercise and diet, stress management and quit smoking programs, marriage counseling, and welfare benefits for women on low incomes. A systematic review of randomized control trials of various lifestyle interventions (e.g. counseling and education in addition to or instead of pharmacological treatments) aimed at reducing cardiovascular disease risk factors showed that strategies which focused on diet, exercise, smoking cessation and alcohol intake reduction were effective for both primary and secondary prevention [56]. However the uptake of lifestyle interventions varies across different social and demographic sub-groups [57]. For example, programs that target mid-aged women differ from those that target men or younger women and the capacity of people from disadvantaged groups to act on lifestyle information may be limited, placing them at greater risk of adverse health consequences. If they are to be effective, broad based community and health promotion programs must address issues for specific socio-demographic and/or socioeconomic sub-groups [57,58]. The results from this study can be used to assist policy-makers by providing information on the types of lifestyle interventions that might be appropriate for mid-aged Australian women, given their varying socioeconomic circumstances. This is particularly important given that mid-life is a time of declining general health for many women. Models 1 and 3 (general heath) suggest that interventions to address obesity and encourage smoking cessation may be effective in bettering the general health of mid-aged women.

Mental health (Models 2 and 4) improved after adjusting for the interaction between SES, the interaction between time and SES, age (measured at baseline) and BMI, marital status, smoking status, ability to manage on income, stress and area of residence, as possible confounders. As noted earlier, this improvement may reflect an easing of psychosocial and occupational factors which impact more heavily on the mental health of women in their late forties and early fifties, than in later years [46]. The association between positive mental health and retirement is also stronger in higher SES occupational groups [26]. However despite evidence of improved mental health in mid-aged women, there is still a need to direct policies towards improving the mental health of specific sub-groups such as lower SES women, smokers, women who are overweight and obese, and women with insufficient income support in retirement.

### Strengths and limitations

There are several strengths of this study. Firstly, the data were from a large representative national sample of mid-aged women who live in cities, outer metropolitan centers and rural and remote areas. Previous studies using this cohort have demonstrated that bias due to attrition is not sufficient to preclude meaningful longitudinal analyses such as the one conducted here [59].

Secondly, we analyzed changes in self-reported general health and mental health separately, although we acknowledge that there may have been associations between these two SF-36 subscales that we did not establish. While there are some exceptions [60], most empirical studies using longitudinal data have employed summary health measures and hence do not make any distinctions between different components of health as we have done here [54,61].

Thirdly, we used longitudinal data that allowed us to track changes in health and other characteristics in the same group of individuals (a mid-aged cohort of women) as they aged from 45 to 64 years. This is a significant phase in a woman’s life, both in terms of changes in general and mental health (in part associated with menopause) and in their socioeconomic circumstances (such as making changes in employment, caring for ageing parents and reaching retirement). Longitudinal studies like this are important because they make it possible to identify and explain changes in health gradients across particular years, and/or the entire life-span. Identification of the life stages at which socioeconomic inequalities stop widening is of policy interest [23]. As subsequent ALSWH survey waves become available it will be possible to undertake analyses covering additional years in the lifetime of these women.

A limitation of the study is that separate adjustments were not made for co-morbidities or particular types of diseases that the women may have had. Previous studies have shown that socioeconomic gradients are substantial for many chronic conditions [23]. It is possible therefore, that in not making these adjustments, we have underestimated associations between SES and health changes. The “healthy survivor” effect may account for the improvement shown here in the mental health of the ALSWH 1946–51 cohort because women with poor mental health may have dropped out of ALSWH [40].

## Conclusions

This Australian study contributes to our current understanding of how SES, socio-demographic factors and health behaviors impact on general and mental health changes in women progressing in years from 45–50 to 59–64.

## Competing interests

The authors declare that they have no competing interests.

## Authors’ contributions

JSW and MC conceptualized the study. JSW undertook the statistical analyses with input from MC. JSW prepared the first draft with input from MC. JB provided advice and guidance throughout the project. All authors read and approved the final draft.
